# Mechanistic investigation of UV-irradiated neem oil against microbial biofilms and A431 skin cancer cells via Bax/Bcl2/caspase-3 modulation

**DOI:** 10.1186/s40643-026-01111-7

**Published:** 2026-09-15

**Authors:** Samy Selim, Aisha M. H. Al-Rajhi, Sulaiman A. Alsalamah, Mutasem S. Almehayawi, Mohammed H. Alruhaili, Hattan S. Gattan, Alaa A. Kashmiry, Manal J. Kiki, Ruba Abdulrahman Ashy, Sahar Abdulaziz Alshareef

**Affiliations:** 1 Department of Clinical Laboratory Sciences, College of Applied Medical Sciences, Jouf University, Sakaka, Saudi Arabia; 2 Department of Biology, College of Science, Princess Nourah bint Abdulrahman University, P.O. Box 84428, 11671 Riyadh, Saudi Arabia; 3 Department of Biology, College of Science, Imam Mohammad Ibn Saud Islamic University (IMSIU), 11623 Riyadh, Saudi Arabia; 4 Department of Emergency Medicine, Faculty of Medicine, King Abdulaziz University and Hospital, Jeddah, Saudi Arabia; 5 Department of Clinical Microbiology and Immunology, Faculty of Medicine, King Abdulaziz University, 21589 Jeddah, Saudi Arabia; 6 Department of Medical Laboratory Sciences, Faculty of Applied Medical Sciences, King Abdulaziz University, Jeddah, Saudi Arabia; 7 Special Infectious Agents Unit, King Fahad Medical Research Center, King Abdulaziz University, Jeddah, Saudi Arabia; 8 Department of Chemistry, Applied College at Khulais, University of Jeddah, Jeddah, Saudi Arabia; 9 Department of Biological Sciences, College of Science, University of Jeddah, 21493 Jeddah, Saudi Arabia; 10 Department of Biology, College of Science and Arts at Khulis, University of Jeddah, 21921 Jeddah, Saudi Arabia

**Keywords:** Neem oil, UV irradiation, Antimicrobial, Antibiofilm, A431 cells, Apoptosis, Cell cycle, Caspase-3

## Abstract

Neem oil (*Azadirachta indica*) possesses antimicrobial and anticancer properties, but its clinical translation is limited by variable chemical profile and suboptimal bioavailability. Ultraviolet (UV) irradiation is a green, non-thermal physical modification technique that can alter phytochemical composition and bioactivity. The study aimed to characterize chemical changes induced by UV irradiation (254 nm, 120 min) in neem oil and evaluate the enhanced antimicrobial, antibiofilm, and anticancer activities against A431 skin cancer cells, including mechanistic insights into cell cycle arrest and *Bax*/*Bcl-2*/*caspase-3* signaling. Crude and UV-irradiated neem oils were analyzed by Fourier transform infrared (FTIR) spectroscopy and Gas chromatography–mass spectrometry (GC–MS). Antimicrobial and antibiofilm activities were tested against five pathogens including Methicillin-resistant *Staphylococcus aureus* (MRSA), *Bacillus subtilis, Escherichia coli*, *Proteus vulgaris* and *Candida albicans*. Antiproliferative activity against A431 cells was assessed by MTT assay, cell cycle analysis (flow cytometry), and qRT-PCR for *Bcl-2*, *Bax*, and *caspase-3*. UV irradiation induced oxidation, cis-to-trans isomerization, and formation of α,*β*-unsaturated carbonyls (FTIR), along with new bioactive compounds: isochiapin B (3.88%) and a steroid derivative (9.40%) by GC–MS. UV-neem oil significantly enhanced antimicrobial activity (inhibition zones up to 30 mm), reduced MIC/MBC up to eightfold (MRSA, MIC: 125 → 15.6 µg/mL), and showed superior biofilm inhibition (91–95% at 25% MBC). UV-neem oil exhibited a lower IC₅₀ (154.4 vs. 179.3 µg/mL), induced stronger S-phase arrest (S-phase reduced to 9.45%), upregulated *Bax* (5.26-fold) and *caspase*-3 (7.65-fold), and downregulated *Bcl-2* (0.53-fold). UV irradiation transforms neem oil into a more potent antimicrobial, antibiofilm, and pro-apoptotic agent against A431 cells via oxidative and isomeric modifications. This green photochemical approach offers a promising strategy to enhance the therapeutic efficacy of plant oils.

**Graphical abstract:**

**Figure:**
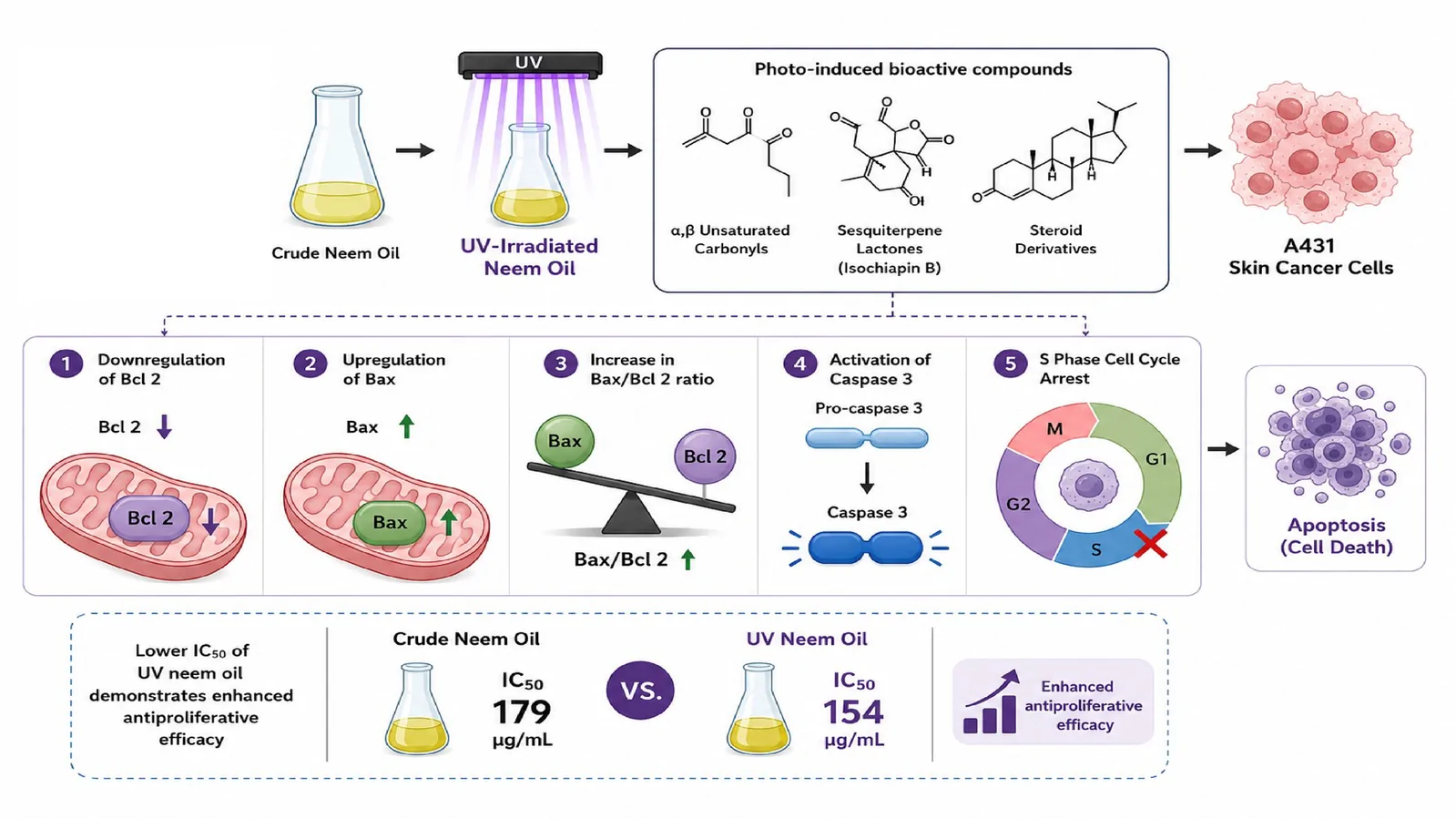

**Supplementary Information:**

The online version contains supplementary material available at https://doi.org/10.1186/s40643-026-01111-7.

## Introduction

Plant-derived oils have attracted considerable attention in recent years owing to their natural origin, biocompatibility, and wide range of biological activities, making them promising candidates for applications in pharmaceutical, medical, cosmetic, and food industries (Alsolami et al. 2023; Qanash et al. 2023a). Their richness in bioactive compounds has encouraged their utilization as sustainable alternatives to synthetic agents in the development of antimicrobial, antioxidant, anti-inflammatory, and therapeutic formulations (Alghonaim 2025; Hammad et al. 2022). Among these natural oils, neem oil, derived from the seeds of *Azadirachta indica* A. Juss. (Meliaceae), has been extensively recognized for its diverse pharmacological properties, including antimicrobial, anti-inflammatory, and anticancer activities (Alzohairy 2016; Wylie and Merrell 2022; Baby et al. 2022). The therapeutic potential of neem oil is primarily attributed to its rich phytochemical composition, comprising terpenoids, limonoids, glycerides, and fatty acids (Batra et al. 2022; Gomes et al. 2025). Despite its bioactivity, clinical translation of crude neem oil is hindered by its variable chemical profile and suboptimal bioavailability (Rinaldi et al. 2017; Segneanu et al. 2025). In recent years, ultraviolet (UV) irradiation has emerged as a green, non-thermal physical modification technique capable of inducing photochemical transformations, such as oxidation, polymerization, and cis–trans isomerization, which can alter the phytochemical landscape and bioactivity of plant oils (Menga et al. 2025; Kibar and Kibar 2025). However, the comparative effects of UV-irradiated versus non-irradiated neem oil on skin cancer cells, particularly the A431 epidermoid carcinoma cell line, remain largely unexplored.

The A431 cell line serves as a well-established model for cutaneous squamous cell carcinoma, a common non-melanoma skin cancer characterized by aggressive local invasion (Corchado-Cobos et al. 2020; Liu and Liu 2024). Emerging evidence highlights that natural compounds exert anticancer effects primarily through the induction of apoptosis, a programmed cell death process tightly regulated by the *Bcl-2* family proteins (pro-apoptotic *Bax* and anti-apoptotic *Bcl-2*) and executioner caspases such as caspase-3 (Wang et al. 2023; Vogler et al. 2025). Moreover, disruption of the cell cycle at specific checkpoints (G0/G1, S, or G2/M) is a critical determinant of antiproliferative efficacy (Selim et al. 2026; Wan et al. 2026). Concurrently, the ability of neem oil to inhibit microbial growth and disrupt biofilm formation—key virulence determinants in chronic infections—adds another layer to its therapeutic versatility (Guchhait et al. 2022; Mahmoud et al. 2024). Nevertheless, the impact of UV irradiation on these multiple bioactivities and the associated molecular mechanisms has not been systematically investigated. Therefore, this study aims to (i) characterize the chemical fingerprints of crude and UV-irradiated neem oil using Fourier Transform Infrared (FTIR) spectroscopy and Gas Chromatography-Mass Spectrometry (GC–MS); (ii) evaluate and compare their antimicrobial and anti-biofilm activities against clinically relevant pathogens; (iii) assess the anticancer activity against A431 cells; and (iv) provide preliminary mechanistic insights into the apoptotic pathway through cell cycle analysis and transcriptional profiling of *Bax*, *Bcl-2*, and *caspase-3*. It is important to note that gene expression analysis at the mRNA level reflects transcriptional regulation and does not directly demonstrate protein-level changes or functional apoptosis. The current study, therefore, provides suggestive rather than conclusive evidence of apoptosis induction, and protein-level validation is required to definitively establish the mechanism.

## Materials and methods

### Neem oil and UV irradiation

Cold-pressed, pure neem oil (*Azadirachta indica*) was obtained from a commercial source (Organic India Pvt. Ltd., Lucknow, India; Lot No. NI-2210). The oil was stored in amber glass bottles at 4 °C until use. For UV irradiation, 10 mL of neem oil was placed in a sterile, uncovered 60 mm borosilicate glass Petri dish (Corning Inc., NY, USA) and exposed to UV-C radiation (254 nm) using a UV crosslinker (SpectroLinker™ XL-1500, Spectronics Corporation, Westbury, NY, USA) equipped with six 15 W tubes. The irradiation was performed at a distance of 10 cm from the source, with an average intensity of 1.2 mW/cm^2^. Based on preliminary time-course experiments (30, 60, 90, 120, and 180 min) evaluating changes in FTIR peak ratios and antimicrobial activity, a 120-min exposure was selected as the optimal time (designated as UV-neem oil). Non-irradiated neem oil served as the control (crude neem oil). Both oil forms were immediately stored at − 20 °C under nitrogen atmosphere to prevent further oxidation (Alsalamah et al. 2025).

### Fourier transform infrared (FTIR) spectroscopy

FTIR spectra of crude and UV-neem oil were recorded using a PerkinElmer Spectrum Two™ FTIR spectrometer (PerkinElmer Inc., Waltham, MA, USA) equipped with a universal attenuated total reflectance (UATR) diamond crystal accessory. Approximately 10 µL of each oil sample was placed directly onto the crystal. Spectra were acquired in the wavenumber range of 4000–400 cm⁻^1^ at a resolution of 4 cm⁻^1^, with 32 scans per sample. Background air spectrum was subtracted automatically. Data were processed using Spectrum IR® software (version 10.6.2). Three independent replicates were analyzed, and representative spectra are reported (Qanash et al. 2023b; Cherif et al. 2025).

### Gas chromatography-mass spectrometry (GC–MS) analysis

Qualitative phytochemical profiling was performed using an Agilent 7890B GC system coupled to an Agilent 5977B single quadrupole MSD (Agilent Technologies, Santa Clara, CA, USA). Samples were prepared by dissolving 20 mg of oil in 1 mL of n-hexane (HPLC grade, Sigma-Aldrich, St. Louis, MO, USA) and filtered through a 0.22 µm PTFE syringe filter (Millipore, Burlington, MA, USA). Separation was achieved on an Agilent HP-5 ms UI capillary column (30 m × 0.25 mm i.d., 0.25 µm film thickness). The oven temperature program was: initial 60 °C (hold 2 min), then ramped at 10 °C/min to 280 °C (hold 10 min). Injector temperature: 250 °C; split ratio 1:20; injection volume 1 µL. Helium (99.999% purity) was used as carrier gas at a constant flow rate of 1.2 mL/min. MS conditions: electron ionization (EI) at 70 eV; ion source temperature 230 °C; quadrupole temperature 150 °C; mass scan range m/z 40–550. Compounds were identified by comparing mass spectra with the NIST 17 spectral library (National Institute of Standards and Technology, Gaithersburg, MD, USA) and by calculating Kovats retention indices relative to a C8–C40 n-alkane standard (Sigma-Aldrich) (Lin et al. 2025). Only compounds with a match factor ≥ 85% and relative abundance ≥ 0.5% of total ion current are reported in Tables 1 and 2. Complete chromatographic data are available from the corresponding author upon request. It is important to acknowledge that GC–MS with library matching provides tentative identification based on mass spectral fragmentation patterns and retention indices. For sesquiterpene lactones such as isochiapin B—which are polar and thermally labile— unequivocal structural confirmation typically requires additional spectroscopic techniques, particularly NMR spectroscopy. The identifications reported here should therefore be considered preliminary and are presented to characterize the major compositional changes induced by UV irradiation.

**Table 1:** Phytochemical composition of crude neem oil identified by GC–MS

RT (min)	Compound	Molecular formula	MW	Class	Area %
28.24	9-Octadecenoic acid (Z)-, methyl ester	C_19_H_36_O_2_	296	Fatty acid ester	1.34
29.34	Oleic Acid	C_18_H_34_O_2_	282	Fatty acid	10.70
34.40	(Z)-3-(Pentadec-8-en-1-yl)phenol	C_21_H_21_O	302	Alkyl phenol	13.90
37.62	Cholestan-3-ol, 2-methylene-	C₂₈H₄₈O	400	Sterol	0.82
39.64	Squalene	C₃₀H₅₀	410	Triterpene	1.93
40.60	Heptacosane	C₂₇H₅₆	380	Alkane	1.80
40.73	Oleic Acid	C₁₈H₃₄O₂	282	Fatty acid	0.67
40.81	Glycidyl oleate	C₂₁H₃₈O₃	338	Epoxidized fatty acid ester	1.03
42.01	Ethyl iso-allocholate	C₂₆H₄₄O₅	436	Bile acid ester (steroid)	0.52
42.49	Heptacosane	C₂₇H₅₆	380	Alkane	2.17
42.83	Stigmasta-5,22-dien-3-ol, acetate	C₃₁H₅₀O₂	454	Phytosterol ester	7.98
44.32	1-Heptatriacotanol	C₃₇H₇₆O	536	Fatty alcohol	1.34
44.83	Oleyl oleate	C₃₆H₆₈O₂	532	Wax ester (fatty acid ester)	55.80

Compounds are listed with retention time (RT), molecular formula, molecular weight, class, and relative abundance (area %)

**Table 2:** Complete phytochemical composition of UV-irradiated neem oil (254 nm, 120 min) identified by GC–MS

RT (min)	Compound	Molecular Formula	MW	Class	Area %
18.27	2,4-Decadienal, (E,E)-	C₁₀H₁₆O	152	Aldehyde	0.62
22.15	n-Hexadecanoic acid	C₁₆H₃₂O₂	256	Saturated fatty acid	3.01
25.42	9,12-Octadecadienoic acid (Z,Z)-, methyl ester	C₁₉H₃₄O₂	294	Fatty acid ester	1.87
28.25	9-Octadecenoic acid (Z)-, methyl ester	C₁₉H₃₆O₂	296	Fatty acid ester	3.01
29.34	Oleic Acid	C₁₈H₃₄O₂	282	Fatty acid	17.93
31.62	9,12-Octadecadienoic acid (Z,Z)-	C₁₈H₃₂O₂	280	Fatty acid	1.22
34.41	(Z)-3-(Pentadec-8-en-1-yl)phenol	C₂₁H₃₄O	302	Alkyl phenol	18.98
36.78	Cholestan-3-ol, 2-methylene-	C₂₈H₄₈O	400	Sterol	0.91
39.64	Squalene	C₃₀H₅₀	410	Triterpene	1.45
40.60	Heptacosane	C₂₇H₅₆	380	Alkane	3.41
40.73	Oleic Acid	C₁₈H₃₄O₂	282	Fatty acid	0.58
40.81	Glycidyl oleate	C₂₁H₃₈O₃	338	Epoxidized fatty acid ester	0.89
42.01	Ethyl iso-allocholate	C₂₆H₄₄O₅	436	Bile acid ester	1.58
42.50	Isochiapin B	C₁₉H₂₂O₆	346	Sesquiterpene lactone	3.88
42.84	Pregna-5,8-diene-3α,11α-diol-20-one diacetate	C₂₅H₃₄O₅	414	Steroid derivative	9.40
43.11	Stigmasta-5,22-dien-3-ol, acetate	C₃₁H₅₀O₂	454	Phytosterol ester	4.12
43.80	(3β,24S)-Stigmast-5-En-3-Ol	C₂₉H₅₀O	414	Phytosterol	2.01
44.32	1-Heptatriacotanol	C₃₇H₇₆O	536	Fatty alcohol	3.25
44.83	trans-13-Octadecenoic acid	C₁₈H₃₄O₂	282	Trans fatty acid	19.23
45.05	Oleyl oleate	C₃₆H₆₈O₂	532	Wax ester	14.31
46.21	Octacosanol	C₂₈H₅₈O	410	Fatty alcohol	0.87
47.58	Sitosterol, (3β)-	C₂₉H₅₀O	414	Phytosterol	0.95

*Identification based on GC–MS mass spectral library matching (NIST 17) and retention index comparison. For sesquiterpene lactones such as isochiapin B, NMR spectroscopy is required for unequivocal structural confirmation

Compounds are listed with retention time (RT), molecular formula, molecular weight, class, and relative abundance (area %). Compounds newly formed or significantly altered by UV treatment are highlighted in bold

### Antimicrobial testing

The preliminary antimicrobial activity of crude and UV-irradiated neem oil was assessed using the agar well diffusion method. Overnight cultures of test strains (*Bacillus subtilis* ATCC 6633, methicillin resistant *Staphylococcus aureus* (MRSA) ATCC 33591, *Escherichia coli* ATCC 25922, *Proteus vulgaris* ATCC 13315, and *Candida albicans* ATCC 10231) were adjusted to 0.5 McFarland standard (1 × 10⁸ CFU/mL) using a DensiCHEK™ Plus turbidimeter (bioMérieux, Marcy-l’Étoile, France). A sterile cotton swab was dipped into each suspension and evenly streaked onto Mueller–Hinton agar (MHA; Becton Dickinson, Franklin Lakes, NJ, USA) for bacteria or Sabouraud dextrose agar (SDA; HiMedia, Mumbai, India) for *C. albicans*. After 10 min drying, wells (8 mm diameter) were punched aseptically with a stainless-steel cork borer (Fisher Scientific, Hampton, NH, USA). Each well received 50 µL of either crude neem oil (500 mg/mL in 1% DMSO), UV-neem oil (500 mg/mL in 1% DMSO), gentamicin (10 µg/well; positive control for bacteria), fluconazole (25 µg/well; positive control for yeast), or 1% DMSO (negative control). Plates were kept at 4 °C for 2 h to permit diffusion, then incubated at 37 °C for 24 h (bacteria) or at 30 °C for 48 h (*C. albicans*). The diameter of the inhibition zone (including the well) was measured with a digital Vernier caliper (Mitutoyo, Kawasaki, Japan). All assays were performed in triplicate (Aldayel and El Semary 2020).

### Minimum inhibitory concentration (MIC)

The MIC was determined using the broth microdilution method following CLSI guidelines M07-A11 and M27-A3. Due to preliminary screening indicating that some test oils required higher concentrations to inhibit growth, a broad concentration range was employed. Crude and UV-irradiated neem oils were dissolved in dimethyl sulfoxide (DMSO; Sigma-Aldrich, St. Louis, MO, USA) to prepare a starting stock of 500 mg/mL (final DMSO concentration in the first well ≤ 1% v/v). Two-fold serial dilutions were prepared in sterile 96-well flat-bottom microtiter plates (Corning Inc., Corning, NY, USA) using MHB (for bacteria) or SDB (for yeast) as diluent, yielding final concentration ranges of 1.95–500 mg/mL for each oil. Each well received 100 µL of the respective oil dilution, followed by 100 µL of microbial suspension adjusted to approximately 5 × 10^5^ colony-forming units (CFU)/mL (equivalent to 0.5 McFarland standard, verified by optical density at 625 nm using a BioPhotometer™ D30 (Eppendorf, Hamburg, Germany). Positive control wells contained medium with inoculum and 0.5% DMSO (oil vehicle) but no oil; negative control wells contained medium alone. Plates were incubated at 37 °C for 18–24 h (bacteria) or at 30 C for 48 h (*C. albicans*) under aerobic conditions in a static incubator (Memmert INE600, Schwabach, Germany). After incubation, 20 µL of resazurin solution (0.01% w/v in sterile distilled water; Sigma-Aldrich) was added to each well, followed by a further 2 h incubation. A color change from blue/purple to pink indicated microbial growth. The MIC was defined as the lowest concentration of each oil that completely inhibited visible growth (no color change from blue to pink). All experiments were performed in triplicate on three separate occasions (Qanash et al. 2023c).

### Minimum bactericidal concentration (MBC) and minimum fungicidal concentration (MFC)

To determine the MBC for bacterial strains and MFC for *C. albicans*, aliquots (10 µL) were taken from all wells showing no visible growth (at or above the MIC) and from the positive control wells. These samples were spot-plated onto MHA (for bacteria) or SDA (for yeast) plates (Becton Dickinson) and incubated at 37 °C for 24 h (bacteria) or 30 °C for 48 h (yeast). The MBC/MFC was defined as the lowest concentration of the tested oil that killed ≥ 99.9% of the initial inoculum, i.e., no colony growth on the agar surface. A reduction of less than 3 log₁₀ CFU/mL compared to the positive control was considered not bactericidal/fungicidal. Each determination was performed in duplicate from three independent experiments (Hashem et al. 2022).

### Antibiofilm assay

Biofilm inhibition was evaluated using crystal violet staining (Altuwaijri et al. 2025). Briefly, microbial suspensions (200 µL of 1 × 10⁶ CFU/mL in MHB supplemented with 1% glucose) were added to 96-well plates and treated with sub-MIC concentrations (25%, 50%, and 75% of MBC or MFC) of each oil. After incubation at 37 °C for 24 h, planktonic cells were removed, wells were washed three times with PBS (pH 7.4, Gibco, Thermo Fisher Scientific, Waltham, MA, USA), and adhered biofilms were fixed with methanol (20 min). Because the tested microorganisms exhibited different susceptibilities to crude and UV-treated neem oils, treatment concentrations were normalized as fractions of the corresponding MBC/MFC rather than using identical absolute concentrations. This approach ensured that each microorganism was exposed to an equivalent relative antimicrobial challenge while avoiding complete microbial killing. The actual concentrations (µg/mL) used for each microorganism are presented in Supplementary Table S1. Staining was performed with 200 µL of 0.1% crystal violet (w/v) for 15 min, followed by solubilization with 33% glacial acetic acid. Absorbance was measured at 595 nm using a Synergy™ H1 microplate reader (BioTek Instruments Inc., Winooski, VT, USA). Percentage inhibition was calculated relative to untreated controls.

### Cell culture and antiproliferative assay

Human epidermoid carcinoma cells (A431; ATCC® CRL-1555™) and Primary human epidermal keratinocytes (HEKa; ATCC® PCS-200–011™) (an epidermoid carcinoma cells (A431; ATCC® CRL-1555™) and primary human epidermal keratinocytes (HEKa; ATCC® PCS-200–011™) (purchased from the American Type Culture Collection, Manassas, VA, USA) and cultured according to the supplier’s recommendations.) were cultured in Dulbecco’s Modified Eagle’s Medium (DMEM; high glucose, L-glutamine, sodium pyruvate; Gibco) supplemented with 10% fetal bovine serum (FBS; heat-inactivated; HyClone™, GE Healthcare, Chicago, IL, USA) and 1% penicillin–streptomycin (10,000 U/mL; Gibco). Cells were maintained at 37 °C in a humidified 5% CO₂ incubator (Heracell™ 150i, Thermo Fisher Scientific). Cell viability was assessed using the 3-(4,5-dimethylthiazol-2-yl)-2,5-diphenyltetrazolium bromide (MTT) assay (Sigma-Aldrich, M5655). A431 cells (1 × 10^4^ cells/well) were seeded in 96-well plates and allowed to attach overnight. Cells were treated with increasing concentrations (31.25–1000 µg/mL) of crude or UV-neem oil (diluted in DMEM with 0.1% DMSO) for 24 h. After treatment, 20 µL of MTT solution (5 mg/mL in PBS) was added per well and incubated for 4 h. Formazan crystals were dissolved in 150 µL DMSO, and absorbance was read at 570 nm (reference 630 nm) using the Synergy™ H1 reader. IC₅₀ values were calculated by non-linear regression using GraphPad Prism 9.5 (GraphPad Software, San Diego, CA, USA). Three independent experiments were performed in quadruplicate (Hasanin et al. 2022).

### Cell cycle analysis by flow cytometry

A431 cells (5 × 10^5^ cells/well in 6-well plates) were treated with the IC₅₀ concentrations of crude or UV-neem oil for 24 h. Untreated cells (0.1% DMSO) served as control. After treatment, cells were harvested by trypsinization (0.25% trypsin–EDTA; Gibco), washed twice with cold PBS, and fixed in 70% ice-cold ethanol at − 20 °C overnight. Fixed cells were washed, resuspended in 500 µL of propidium iodide (PI) staining solution (50 µg/mL PI, 100 µg/mL RNase A, 0.1% Triton X-100 in PBS; all from Sigma-Aldrich), and incubated for 30 min at 37 °C in the dark. DNA content was analyzed using a BD FACSCanto™ II flow cytometer (BD Biosciences, San Jose, CA, USA) equipped with a 488 nm argon laser. At least 20,000 events were acquired per sample. Cell cycle distribution (G0/G1, S, G2/M phases) was determined using FlowJo™ v10.8 (BD Biosciences) (Daddiouaissa et al. 2019).

#### Gene expression analysis by quantitative real-time PCR (qRT-PCR)

Total RNA was extracted from A431 cells (treated with IC₅₀ concentrations of oils for 24 h) using the RNeasy Plus Mini Kit (Qiagen, Hilden, Germany; Cat. No. 74134) according to the manufacturer’s protocol, including on-column DNase digestion. RNA purity and concentration were assessed via A₂₆₀/₂₈₀ ratio (≥ 1.9) using a NanoDrop™ One spectrophotometer (Thermo Fisher Scientific). Complementary DNA (cDNA) was synthesized from 1 µg of total RNA using the High-Capacity cDNA Reverse Transcription Kit (Applied Biosystems, Foster City, CA, USA; Cat. No. 4368814) with random hexamers in a T100™ thermal cycler (Bio-Rad Laboratories, Hercules, CA, USA). Quantitative PCR was performed using PowerUp™ SYBR™ Green Master Mix (Applied Biosystems, Cat. No. A25742) on a CFX96 Touch™ Real-Time PCR Detection System (Bio-Rad). Primer sequences (synthesized by Eurofins Genomics, Louisville, KY, USA) were:

*Bax* (forward: 5′-GGG TGG TTG CCC TCT TCT ACT-3′; reverse: 5′-TCC TGT CCA CGT CAG CAA TCA-3′).

*Bcl-2* (forward: 5′-ATC GCC CTG TGG ATG ACT GAG-3′; reverse: 5′-GCC AGG AGA AAT CAA ACA GAG GC-3′).

*Caspase-3* (forward: 5′-GCA GCC AAG TGA GGA GGA TCA-3′; reverse: 5′-CTC GGC ATG GTG CCC TGT TCA-3′).

*GAPDH* (housekeeping; forward: 5′-GAG TCA ACG GAT TTG GTC GT-3′; reverse: 5′-TTG ATT TTG GAG GGA TCT CG-3′).

Reaction mixture (10 µL total) contained: 5 µL SYBR Green Master Mix, 0.5 µM each primer, 2 µL cDNA (50 ng equivalent), and nuclease-free water. Cycling conditions: 50 °C for 2 min, 95 °C for 2 min, then 40 cycles of 95 °C for 15 s and 60 °C for 60 s, followed by melt curve analysis (65–95 °C, 0.5 °C increments). Each sample was run in triplicate. Relative gene expression was calculated using the 2⁻ΔΔCᵀ method (Abouzed et al. 2021) normalized to GAPDH and expressed as fold change relative to untreated control. It is important to acknowledge that qRT-PCR measures mRNA transcript levels, which reflect transcriptional regulation. These data do not directly measure protein expression, post-translational modifications (e.g., caspase-3 cleavage), or functional apoptotic activity. Therefore, the qRT-PCR findings in this study provide mechanistic evidence at the transcriptional level and require validation at the protein level (e.g., Western blot for Bax, Bcl-2, cleaved caspase-3) and functional level (e.g., Annexin V-FITC/PI flow cytometry, TUNEL assay) to conclusively confirm apoptosis induction.

#### Statistical analysis

All experiments were performed in at least three independent biological replicates (*n* = 3), and data are presented as mean ± standard deviation (SD). Normality was assessed using Shapiro–Wilk test. Comparisons between two groups were performed using two-tailed Student’s *t*-test. Multiple group comparisons were analyzed by one-way ANOVA followed by Tukey’s post-hoc test (for parametric data) or Kruskal–Wallis with Dunn’s test (for non-parametric). IC₅₀ values were calculated using non-linear regression (log(inhibitor) vs. normalized response). Statistical significance was set at *p* < 0.05. All analyses were conducted using GraphPad Prism 9.5.0 (GraphPad Software, San Diego, CA, USA).

## Results and discussion

### Photochemical functional group modifications by FTIR

**Fig. 1:**
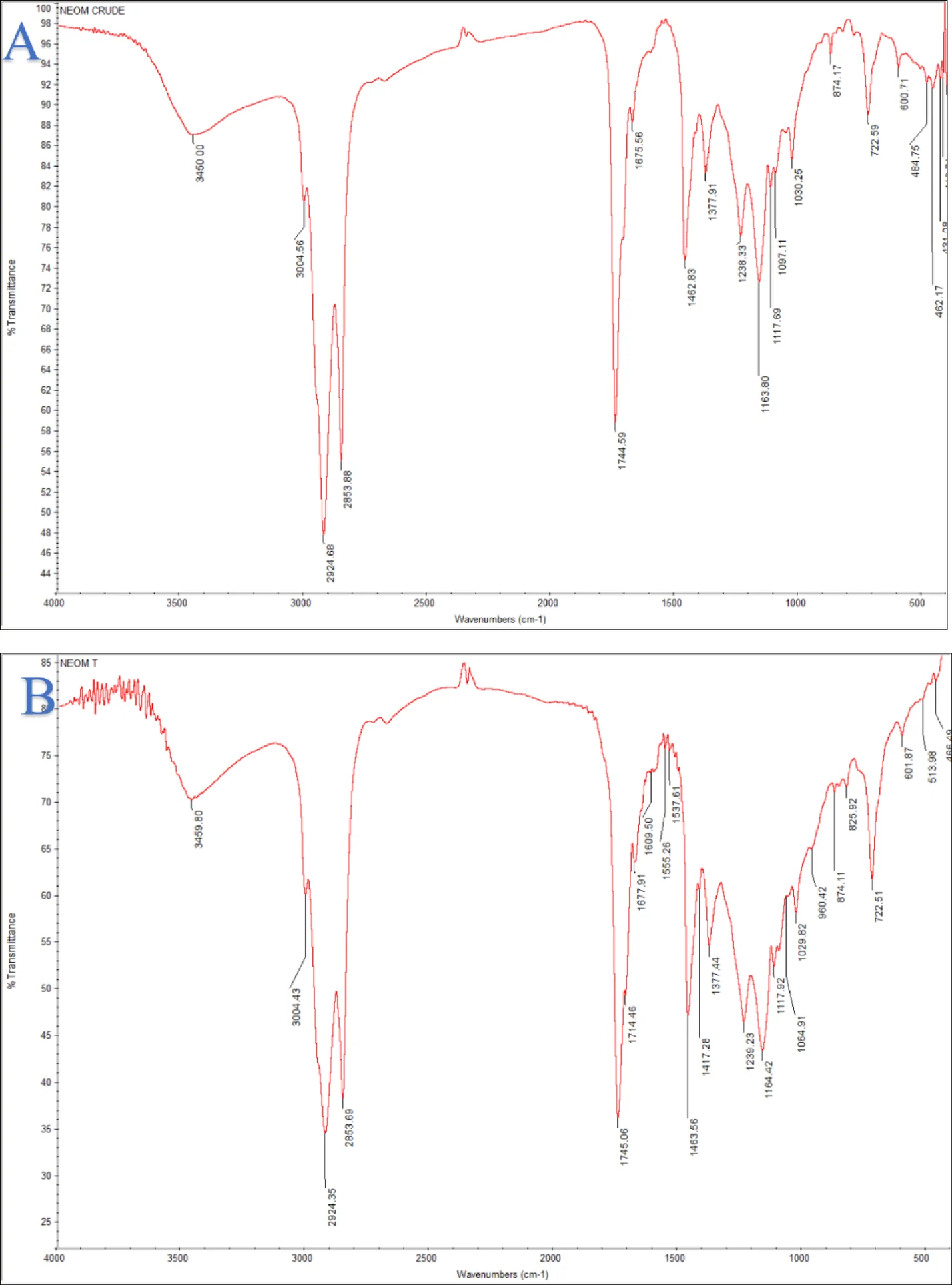
FTIR spectra of **A** crude neem oil and **B** UV-irradiated neem oil (254 nm, 120 min). Major absorption bands are labeled with their respective wavenumbers (cm⁻^1^). The spectra demonstrate photochemically induced modifications in functional groups following UV exposure

FTIR spectra of crude and UV-irradiated (254 nm, 120 min) neem oil revealed distinct photochemically induced modifications in functional groups. Crude neem oil exhibited characteristic bands at 3450.00 cm⁻^1^ (broad O–H stretching of hydroxyl groups/residual moisture), 2924.68 and 2853.88 cm⁻^1^ (asymmetric/symmetric C–H stretching of aliphatic CH₂/CH₃), 1745.56 cm⁻^1^ (ester C = O of triglycerides), 1462.83 cm⁻^1^ (CH₂ scissoring), 1377.91 cm⁻^1^ (CH₃ bending), 1238.33 and 1177.19 cm⁻^1^ (C–O–C of ester linkages), 1007.11 cm⁻^1^ (primary alcohol C–O), and 872.59 cm⁻^1^ (=C–H bending of cis-alkenes) (Fig. 1A). UV-irradiated neem oil showed significant spectral changes: emergence of a band at 3044.43 cm⁻^1^ (=C–H stretching of vinyl/epoxide groups), a new prominent band at 1643.56 cm⁻^1^ indicative of α,*β*-unsaturated carbonyls/enones from photo-oxidation, appearance of 1609.50 and 1555.26 cm⁻^1^ (conjugated diene/aromatic C=C stretching), and a band at 960.42 cm⁻^1^ confirming cis-to-trans isomerization of double bonds. Additionally, the fingerprint region displayed bands at 1417–1428 cm⁻^1^ (C–O–H of oxidized species), 1164.42/1172.93 cm⁻^1^ (secondary alcohol/ether C–O), and 722.51 cm⁻^1^ (aliphatic polymerization). The broad O–H band at 3450 cm⁻^1^ was markedly reduced, suggesting dehydration or hydrogen bonding disruption (Fig. 1B). These photochemical transformations—oxidation, conjugation, isomerization, and partial polymerization—are consistent with previous reports on UV-treated plant oils (Mlakić et al. 2023; Shaikh et al. 2026). The formation of α,β-unsaturated carbonyls (1643 cm⁻^1^) and conjugated dienes (1609 cm⁻^1^) is mechanistically critical, as such electrophilic species can react with cellular thiol groups in microbial enzymes and cancer cell signaling proteins, potentially enhancing antimicrobial and pro-apoptotic activities. The cis-to-trans isomerization (960 cm⁻^1^) and emergence of ether linkages (1164 cm⁻^1^) reflect UV-induced free radical reactions and crosslinking, similar to observations in UV-irradiated *Aloe vera* oil (Alsalamah et al. 2025). Importantly, the preservation of major ester carbonyl bands indicates that the triglyceride backbone remains largely intact, ensuring structural stability while introducing reactive oxygenated moieties. These modifications likely increase oil polarity and membrane penetrability, addressing the bioavailability limitations of crude neem oil (Segneanu et al. 2025; Murru et al. 2021; Wunderling et al. 2023). Therefore, FTIR analysis confirms that 120 min UV-C irradiation effectively generates a chemically modified neem oil with enhanced electrophilic character, providing a molecular basis for its improved biological activities.

### UV-induced phytochemical reconfiguration by GC–MS

**Fig. 2:**
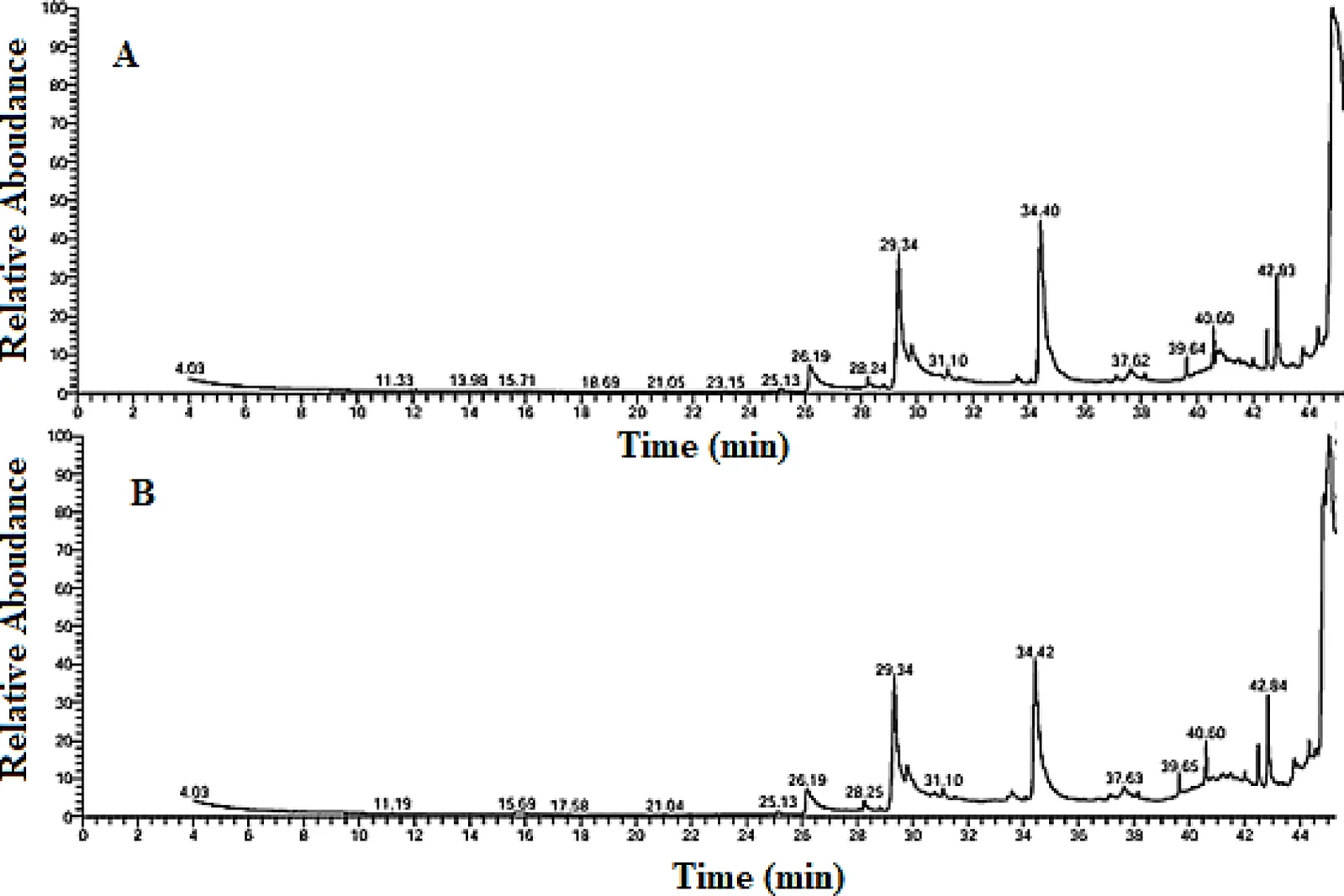
Total ion chromatograms of (**A**) crude neem oil and (**B**) UV-irradiated neem oil (254 nm, 120 min). Peaks are labeled with retention times (RT, min). UV irradiation induced substantial qualitative and quantitative changes in the phytochemical profile. Compound identifications are based on GC–MS mass spectral library matching (NIST 17) and are considered tentative. NMR spectroscopy would be required for unequivocal structural confirmation

GC–MS profiling revealed marked qualitative and quantitative differences between crude and UV-irradiated neem oil, corroborating the FTIR observations of photo-oxidation, isomerization, and conjugation. In crude neem oil, the predominant component was oleyl oleate (a wax ester, 55.80 area%), followed by the alkyl phenol (Z)-3-(pentadec-8-en-1-yl)phenol (13.90%) and oleic acid (10.70%). Minor constituents included squalene (1.93%), a phytosterol ester (7.98%), and an epoxidized fatty acid ester (glycidyl oleate, 1.03%). The presence of a small amount of glycidyl oleate suggests baseline autoxidation even in crude oil (Fig. 2A and Table 1). The complete GC–MS dataset for UV-irradiated neem oil is presented in Table 2, which includes all detectable compounds (≥ 0.5% area) and allows comprehensive verification of compositional changes. Key transformations are highlighted in Table 3 for clarity. UV irradiation induced profound changes. The most striking difference was the dramatic reduction of oleyl oleate from 55.80 to 14.31%, accompanied by a concurrent increase in free oleic acid (from 10.70 to 17.93%) and the appearance of trans-13-octadecenoic acid (19.23% area). This cis-to-trans isomerization of double bonds directly supports the FTIR finding at 960 cm^−1^ and aligns with (Kibar and Kibar 2025), who reported UV-induced geometric isomerization in unsaturated fatty acids. The generation of trans fatty acids is mechanistically significant, as trans isomers can adopt extended conformations that alter membrane fluidity and may enhance bioactivity. Notably, UV treatment promoted the formation of novel oxygenated and rearranged terpenoids. The appearance of a compound tentatively identified as isochiapin B (3.88%), a sesquiterpene lactone, and pregna-5,8-diene-3α,11α-diol-20-one diacetate (9.40%), a steroid derivative, indicates UV-induced oxidative cyclization and rearrangement of triterpene precursors. The identification of isochiapin B is based on GC–MS mass spectral library matching (NIST 17) and retention index comparison. While this approach is consistent with previous literature reports identifying isochiapin B by GC–MS, it constitutes tentative identification. NMR spectroscopy would be required for definitive structural confirmation. These compounds are absent in crude oil and represent photochemical neo-formation. Additionally, the phytosterol (3β,24S)-stigmast-5-en-3-ol (2.01%) emerged, while the original stigmasterol acetate decreased, suggesting deacetylation and structural modification. The alkyl phenol content increased from 13.90% to 18.98%, possibly due to UV-induced decarboxylation or side-chain cleavage of phenolic precursors. The saturated fatty acid n-hexadecanoic acid (palmitic acid) appeared only after UV treatment (3.01%), indicating partial saturation or fragmentation of unsaturated chains. The increase in heptacosane (from 1.80% to 3.41%) and fatty alcohol (1.34% to 3.25%) points to UV-mediated reduction or decarboxylation reactions (Fig. 2B and Table 2). From a bioactivity perspective, the UV-induced generation of α,*β*-unsaturated carbonyls (FTIR band at 1643 cm^−1^) is now chemically substantiated by the formation of sesquiterpene lactones (isochiapin B) and steroid enones, both known to act as Michael acceptors that covalently modify cellular thiols. The present results partially agree with those obtained by Dhowtal et al. (Dhowtal et al. 2025), who reported that UV-C exposure affected the chemical composition and functional properties of beetroot extract. The substantial increase in free fatty acids and trans isomers likely improves membrane penetration, addressing the bioavailability limitations of crude neem oil (Segneanu et al. 2025; Hamadou et al. 2020). Moreover, the retention of the alkyl phenol (a known antimicrobial and anticancer agent) at higher relative abundance, combined with newly formed electrophilic species, provides a mechanistic rationale for the enhanced antimicrobial, antibiofilm, and pro-apoptotic activities observed in this study. Our findings indicated that GC–MS analysis confirms that UV-C irradiation (120 min) fundamentally reshapes the phytochemical landscape of neem oil—reducing inert wax esters, liberating bioactive free fatty acids, generating trans isomers, and inducing the de novo synthesis of reactive terpenoid lactones and steroids. These chemical changes are fully consistent with the FTIR spectral data and collectively underpin the enhanced biological activities reported below.

**Table 3:** Key compositional changes in neem oil induced by UV-C irradiation (254 nm, 120 min)

Compound Class	Crude Oil (Area %)	UV-Oil (Area %)	Change	Interpretation
Wax ester (oleyl oleate)	55.80	14.31	↓** 41.49%**	Hydrolysis to free fatty acids
Free fatty acids (oleic acid)	10.70	17.93	↑** 7.23%**	Increased membrane penetrability
Trans fatty acid (trans-13-octadecenoic acid)	0.00	19.23	↑** 19.23%**	UV-induced cis → trans isomerization
Alkyl phenol	13.90	18.98	↑** 5.08%**	Decarboxylation/side-chain cleavage
Sesquiterpene lactone (isochiapin B)	0.00	3.88	↑** 3.88%**	UV-induced oxidative cyclization
Steroid derivative	0.00	9.40	↑** 9.40%**	UV-induced rearrangement
Phytosterol (free)	0.00	2.01	↑** 2.01%**	Deacetylation of phytosterol ester
Saturated fatty acid (palmitic acid)	0.00	3.01	↑** 3.01%**	Partial saturation
Alkane (heptacosane)	1.80	3.41	↑** 1.61%**	UV-mediated reduction
Fatty alcohol	1.34	3.25	↑** 1.91%**	UV-mediated reduction

Compounds are grouped by class to highlight photochemical transformations

### Enhanced antimicrobial spectrum by agar diffusion testing

**Fig. 3:**
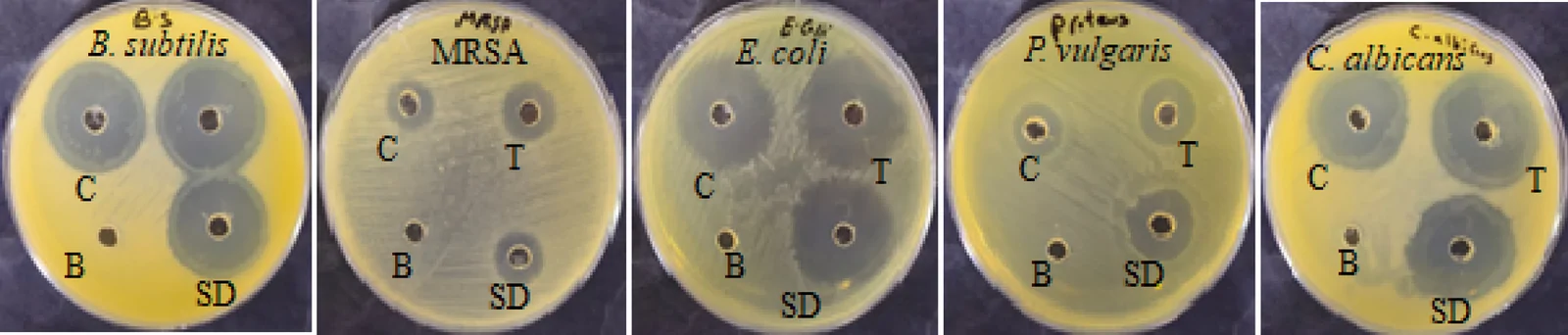
Antimicrobial activity of crude and UV-irradiated neem oil against tested microorganisms by agar well diffusion. Representative plates showing inhibition zones for *B. subtilis*, MRSA, *E. coli*, *P. vulgaris*, and *C. albicans*. Wells: (B) Blank, (C) crude neem oil, (T) UV-neem oil, (SD) positive control (gentamicin for bacteria, fluconazole for yeast)

UV-irradiated neem oil produced significantly larger inhibition zones than crude oil against all tested strains, indicating enhanced antimicrobial potency. The most pronounced increases were observed against *E. coli* (25 to 29 mm, *p* < 0.001), *C. albicans* (25 to 29 mm, *p* < 0.001), and MRSA (12 to 15 mm, *p* < 0.01). Notably, the activity of UV-neem oil against MRSA was not statistically different from gentamicin (15 mm, *p* > 0.05), while against *C. albicans* it significantly exceeded fluconazole (29 vs. 27 mm, #*p* < 0.05) (Table 4 & Fig. 3). These results directly correlate with the chemical modifications identified by FTIR and GC–MS. In addition, our antimicrobial results are in agreement with the study of Rahman et al., (Rahman et al. 2020), which showed that UV-C irradiation significantly enhanced the antimicrobial activities of orange oil. These observations suggest that UV-C treatment may improve the biological efficacy of plant oils through modulation of their bioactive constituent. The UV-induced formation of α,*β*-unsaturated carbonyls (FTIR band at 1643 cm^−1^) and conjugated dienes (1609 cm^−1^) generates electrophilic species capable of alkylating critical microbial enzymes, such as penicillin-binding proteins and DNA gyrase (Wylie and Merrell 2022; Bao et al. 2024). The increase in free fatty acids (oleic acid from 10.7 to 17.9% by GC–MS) and trans-13-octadecenoic acid (0% to 19.2%) likely disrupts bacterial membrane integrity, facilitating greater diffusion of active compounds. Furthermore, the UV-generated sesquiterpene lactone isochiapin B (3.88%) and the steroid derivative (9.40%) are known to inhibit fungal ergosterol biosynthesis, explaining the enhanced activity against *C. albicans* (Mahmoud et al. 2024; Contreras Martínez et al. 2022).

**Table 4:** Inhibition zone diameters (mm) of crude and UV-irradiated neem oil against tested microorganisms by agar well diffusion

Microorganism	Inhibition zone (mm)		
	Crude neem oil	UV-neem oil	Control
*B. subtilis*	28 ± 0.4	30 ± 0.5*	29 ± 0.3
MRSA	12 ± 0.8	15 ± 0.3**	15 ± 0.5
*E. coli*	25 ± 0.2	29 ± 0.5***	29 ± 0.8
*P. vulgaris*	14 ± 0.9	16 ± 0.2*	19 ± 0.1#
*C. albicans*	25 ± 0.1	29 ± 0.4***	27 ± 0.9

Values are mean ± SD (*n* = 3). Control: gentamicin (bacteria) or fluconazole (yeast) at 1.0 mg/mL. Statistical comparisons: *p < 0.05, **p < 0.01, ***p < 0.001 vs. crude oil (paired t-test). #*p* < 0.05 vs. control

### Potentiation of minimum inhibitory /bactericidal/fungicidal concentration (MIC/MBC/MFC)

UV irradiation dramatically reduced both MIC and MBC/MFC values across all microorganisms, with the most striking improvement against MRSA (MIC: 125 to 15.62 µg/mL, eightfold, *p* < 0.001; MBC: 250 to 31.25 µg/mL, eightfold, *p* < 0.001). Against *E. coli*, the MBC decreased eightfold (125 to 15.62 µg/mL, *p* < 0.001), indicating that UV-neem oil is particularly effective against Gram-negative bacteria despite their outer membrane barrier. The MBC/MIC ratios of 2 for all UV-neem oil treatments confirm a bactericidal/fungicidal mode of action (ratio ≤ 4), whereas crude oil showed MBC/MIC ratios of 2–8, indicating occasional bacteriostatic behavior (Tables 5, 6). These quantitative improvements are mechanistically explained by the GC–MS data. The sharp reduction in inert wax ester (oleyl oleate from 55.8% to 14.3%) and the corresponding increase in free oleic acid (10.7% to 17.9%) and trans-13-octadecenoic acid (0% to 19.2%) enhance the oil’s ability to penetrate microbial membranes. Trans fatty acids adopt extended conformations that increase membrane fluidity and permeability, facilitating the entry of other bioactive compounds (Kibar and Kibar 2025; Yu et al. 2018). The UV-induced generation of isochiapin B (3.88%), a sesquiterpene lactone bearing an α-methylene-γ-lactone moiety, is particularly significant; such structures are known to covalently modify the active site cysteine residues of bacterial enoyl-ACP reductase (FabI) and fungal lanosterol 14α-demethylase (CYP51), leading to potent bactericidal and fungicidal effects (Alzohairy 2016; Cheeran and Munuswamy-Ramanujam 2020). The presence of the steroid derivative pregna-5,8-diene-3α,11α-diol-20-one diacetate (9.40%) further supports the fungicidal activity, as steroid analogs can disrupt ergosterol biosynthesis and membrane integrity in *C. albicans* (Wang et al. 2023; Desai et al. 2023). Thus, these chemical transformations explain the enhanced potency and the shift toward a purely bactericidal profile.

**Table 5:** Minimum inhibitory concentration (MIC) of crude and UV-neem oil (µg/mL)

Microorganism	Crude MIC	UV-neem MIC	Fold reduction	*p*-value
*B. subtilis*	31.25 ± 0.1	15.62 ± 0.1	twofold	*p < 0.05
MRSA	125 ± 0.2	15.62 ± 0.2	eightfold	***p < 0.001
*E. coli*	15.62 ± 0.1	7.8 ± 0.1	twofold	*p < 0.05
*P. vulgaris*	31.25 ± 0.3	15.62 ± 0.1	twofold	*p < 0.05
*C. albicans*	31.25 ± 0.1	15.62 ± 0.1	twofold	*p < 0.05

Values are means of three independent replicates (SD < 5% of mean). Statistical comparison: MIC values were log₂-transformed and compared by paired *t*-test; *p < 0.05, **p < 0.01, ***p < 0.001

**Table 6:** Minimum bactericidal/fungicidal concentration (MBC/MFC) of crude and UV-neem oil (µg/mL)

Microorganism	Crude MBC or MFC	UV-neem MBC or MFC	Fold reduction	p-value	MBC or MFC /MIC ratio (UV-neem)
*B. subtilis*	62.5	31.25	twofold	*p < 0.05	2
MRSA	250	31.25	eightfold	***p < 0.001	2
*E. coli*	125	15.62	eightfold	***p < 0.001	2
*P. vulgaris*	62.5	31.25	twofold	*p < 0.05	2
*C. albicans*	125	31.25	fourfold	**p < 0.01	2

Values are means of three independent replicates (SD < 5% of mean). Statistical comparison: MIC values were log₂-transformed and compared by paired *t*-test; *p < 0.05, **p < 0.01, ***p < 0.001

### Superior biofilm eradication at Sub-MBC levels

**Fig. 4:**
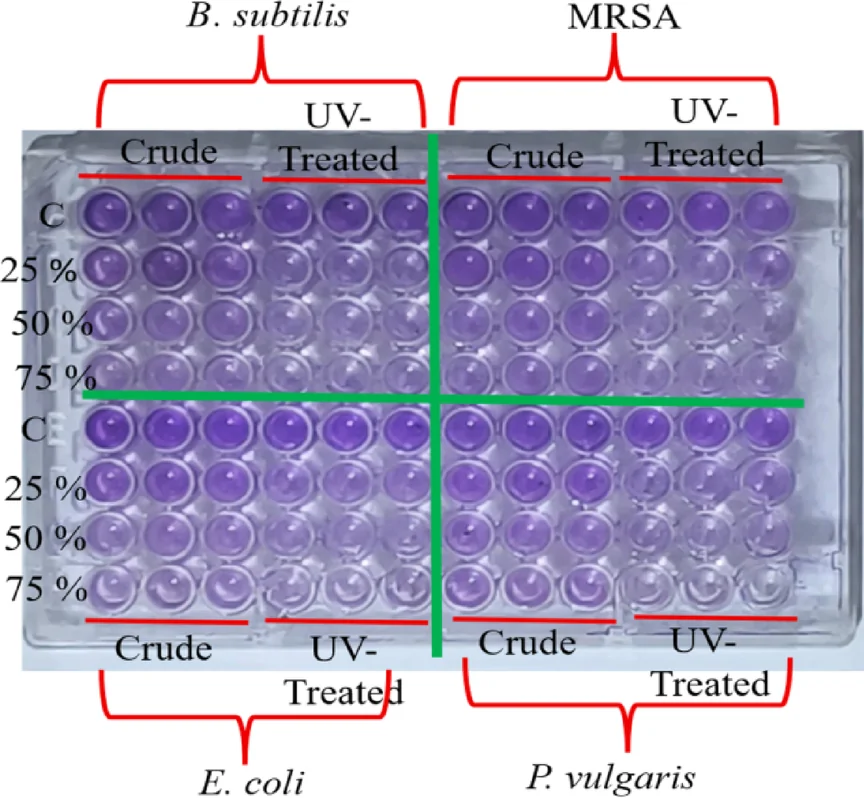
Antibiofilm activity of crude and UV-neem oil visualized by crystal violet staining in 96-well plates against *B. subtilis*, MRSA, *E. coli*, and *P. vulgaris*. Representative image showing biofilm formation (purple) after treatment with 25%, 50%, and 75% of MBC. Reduced purple intensity indicates biofilm inhibition, with UV-neem oil showing superior activity

UV-neem oil exhibited significantly superior biofilm inhibition compared to crude oil at all tested concentrations. At the lowest concentration (25% of MBC), UV-neem oil achieved 71.7% (*B. subtilis*), 91.4% (MRSA), 86.0% (*E. coli*), and 79.4% (*P. vulgaris*), whereas crude oil gave only 26.6–46.7% inhibition (*p* < 0.001 for all). The observed high inhibition rates (91–95%) at sub-MBC concentrations are mechanistically plausible given the multi-target nature of UV-neem oil’s biofilm-disrupting activity. The UV-induced generation of free fatty acids (oleic acid + 7.2%, trans-octadecenoic acid + 19.2%) is known to inhibit exopolysaccharide (EPS) synthesis by downregulating biofilm-associated operons (*pgaABCD* in *E. coli*, *icaADBC* in MRSA) at concentrations well below bactericidal thresholds. Additionally, the newly formed sesquiterpene lactone isochiapin B (3.88%) and steroid derivative (9.40%) act as quorum sensing (QS) inhibitors, disrupting cell-to-cell communication and biofilm maturation without necessarily killing planktonic cells. The alkyl phenol (Z)-3-(pentadec-8-en-1-yl)phenol (18.98%) further contributes by inhibiting surface adhesion and efflux pumps. Thus, the potent antibiofilm activity observed at sub-MBC concentrations reflects true biofilm disruption rather than bactericidal effects, consistent with the known mechanisms of these phytochemical classes. Against MRSA at 25% MBC, UV-neem oil (91.4%) was statistically equivalent to crude oil at 75% MBC (91.8%, *p* > 0.05), representing a three-fold increase in antibiofilm potency (lower concentration achieving same effect). Notably, UV-neem oil maintained > 85% inhibition even at 25% MBC against MRSA and *E. coli*, indicating that the compound acts primarily by disrupting biofilm assembly rather than merely killing planktonic cells (Table 7 and Fig. 4). Since the tested microorganisms exhibited markedly different susceptibilities to crude and UV-treated neem oils, antibiofilm activity was evaluated using normalized sub-MBC/MFC concentrations (25%, 50%, and 75% of the corresponding MBC or MFC) rather than identical absolute concentrations. This normalization ensured that each microorganism was exposed to an equivalent fraction of its own bactericidal or fungicidal threshold, allowing biologically meaningful comparison of antibiofilm efficacy while minimizing bias arising from differences in intrinsic antimicrobial susceptibility (Supplementary Table S1). The importance of this normalization is illustrated by the substantial variation in MBC/MFC values among the tested microorganisms. For example, the MBC of crude neem oil against MRSA was 250 µg/mL, whereas the MBC of UV-treated neem oil against *E. coli* was only 15.62 µg/mL. Therefore, if a fixed concentration of 50 µg/mL had been used for all microorganisms, it would represent only 20% of the MBC for MRSA, producing relatively weak antimicrobial pressure, whereas the same concentration would exceed three times the MBC for *E. coli*, leading to extensive bacterial killing. Under these circumstances, any apparent reduction in biofilm biomass would predominantly reflect loss of viable cells rather than genuine inhibition of biofilm formation. Expressing treatment concentrations as fractions of the corresponding MBC/MFC therefore provides a more scientifically valid comparison of antibiofilm activity across microorganisms with different intrinsic susceptibilities.

**Table 7:** Biofilm inhibition (%) of crude and UV-neem oil at sub-MBC concentrations

Microorganism	Conc (% MBC)	Crude neem oil (% inhibition)	UV-neem oil (% inhibition)	*p-*value (crude vs. UV)
*B. subtilis*	75	89.2 ± 0.4	92.1 ± 0.6	**p < 0.01
	50	68.5 ± 0.7	85.7 ± 0.2	***p < 0.001
	25	40.9 ± 0.7	71.7 ± 0.4	***p < 0.001
*MRSA*	75	91.8 ± 0.6	95.8 ± 0.4	*p < 0.05
	50	68.5 ± 0.7	93.5 ± 0.6	***p < 0.001
	25	40.9 ± 0.7	91.4 ± 0.9	***p < 0.001
*E. coli*	75	90.9 ± 0.3	93.4 ± 0.9	*p < 0.05
	50	82.3 ± 0.3	90.5 ± 0.5	**p < 0.01
	25	46.7 ± 0.7	86.0 ± 0.4	***p < 0.001
*P. vulgaris*	75	82.7 ± 0.3	94.7 ± 0.2	***p < 0.001
	50	73.5 ± 0.2	88.5 ± 0.4	***p < 0.001
	25	26.6 ± 1.9	79.4 ± 0.8	***p < 0.001

Values are mean ± SD (*n* = 3). Statistical significance between crude and UV-neem at each concentration is indicated *p < 0.05, **p < 0.01, ***p < 0.001

The exceptional antibiofilm activity of UV-neem oil is mechanistically linked to the chemical modifications identified by GC–MS. Biofilm formation depends on initial bacterial adhesion, exopolysaccharide (EPS) production, and quorum sensing (QS). The UV-induced increase in free fatty acids (oleic acid + 7.2%, trans-octadecenoic acid + 19.2%) is known to inhibit EPS synthesis by downregulating the *pgaABCD* operon in *E. coli* and the *icaADBC* locus in MRSA (Guchhait et al. 2022; Han et al. 2024). More importantly, the newly formed sesquiterpene lactone isochiapin B (3.88%) and the steroid derivative (9.40%) are potent quorum sensing inhibitors. Sesquiterpene lactones can interfere with the Agr two-component system in MRSA, reducing the production of virulence factors and biofilm-associated proteins (Mahmoud et al. 2024; Mazur and Masłowiec 2022). Steroid derivatives have been shown to antagonize the LuxS/AI-2 QS system in Gram-negative bacteria, thereby preventing biofilm maturation. The alkyl phenol (Z)-3-(pentadec-8-en-1-yl)phenol, which increased from 13.9% to 18.98% after UV, further contributes by disrupting membrane potential and inhibiting efflux pumps, leading to reduced surface adhesion and increased biofilm dispersal (Juszczuk-Kubiak 2024). The dramatic reduction in biofilm formation even at 25% of MBC (i.e., well below the bactericidal concentration) confirms that UV-neem oil acts as a true antibiofilm agent—a highly desirable property for treating chronic implant-associated infections where biofilms are refractory to conventional antibiotics.

### Increased antiproliferative potency against A431 cells (MTT)

**Fig. 5:**
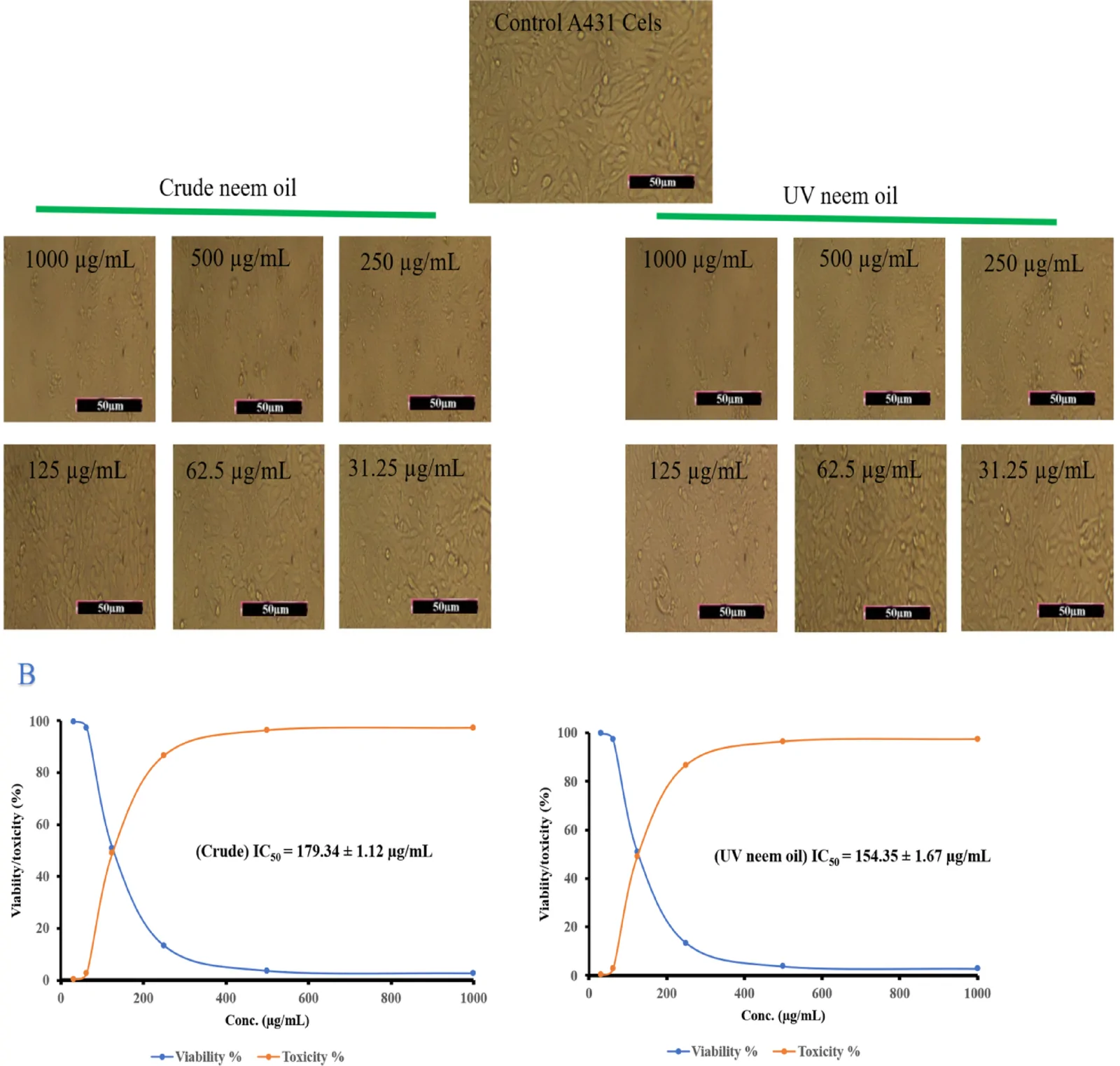
**A** Inverted microscope images of A431 cells after 24 h treatment with increasing concentrations (31.25–1000 µg/mL) of crude neem oil and UV-neem oil. Morphological changes (cell shrinkage, rounding, detachment) indicative of apoptosis are more pronounced in UV-neem oil-treated cells, especially at lower concentrations, consistent with its lower IC₅₀ (Scale bar 50 µm). **B** Dose–response curves of crude and UV-neem oil against A431 cells after 24 h treatment (MTT assay). Cells were treated with increasing concentrations (31.25–1000 µg/mL). Viability (%) is expressed relative to untreated control (0.1% DMSO). Data are mean ± SD (n = 3). IC₅₀ values: crude = 179.34 ± 1.12 µg/mL, UV-neem = 154.35 ± 1.67 µg/mL. UV-neem oil shows a leftward shift of the curve, indicating enhanced antiproliferative potency

The antiproliferative activity of neem oil against A431 cells was significantly enhanced following UV irradiation. Compared with the crude oil, UV-treated neem oil exhibited a 1.16-fold reduction in the IC₅₀ value (from 179.34 to 154.35 µg/mL, *p* < 0.001), demonstrating that UV-induced photochemical modification effectively potentiated its anticancer activity (Fig. 5 and Table 8). Although these data are not included in Table 8, both crude and UV-irradiated neem oils exhibited relatively low cytotoxicity toward primary human epidermal keratinocytes, with IC₅₀ values of 310.66 ± 1.25 and 321.50 ± 3.66 µg/mL, respectively. Notably, the substantially higher IC₅₀ values observed in normal keratinocytes than in A431 cancer cells indicate a favorable therapeutic window, suggesting that UV irradiation enhances the selective anticancer efficacy of neem oil while preserving its biocompatibility toward normal skin cells. This improvement correlates directly with the UV-induced chemical changes documented by FTIR and GC–MS: the generation of α,*β*-unsaturated carbonyls (1643 cm⁻^1^), conjugated dienes (1609 cm⁻^1^), and the de novo synthesis of reactive terpenoids such as isochiapin B (sesquiterpene lactone, 3.88%) and a steroid derivative (9.40%). These electrophilic species are known to induce apoptosis in cancer cells by covalently modifying cysteine residues in key regulatory proteins (Zhao et al. 2023, 2022). Furthermore, the increase in free fatty acids (oleic acid + 7.2%, trans-octadecenoic acid + 19.2%) and the alkyl phenol (from 13.9 to 18.98%) contributes to membrane disruption and mitochondrial dysfunction, lowering the threshold for apoptotic initiation.

**Table 8:** IC₅₀ values (µg/mL) of crude and UV-neem oil against A431 human epidermoid carcinoma cells after 24 h treatment (MTT assay)

Treatment	IC₅₀ (µg/mL)	95% CI	p-value
Crude neem oil	179.34 ± 1.12	[176.9 – 181.8]	–
UV-neem oil	154.35 ± 1.67	[150.7 – 158.0]	***p < 0.001

Values are mean ± SD (*n* = 3). ***p < 0.001 vs. crude (unpaired t-test)

### S-Phase cell cycle arrest demonstrated by flow cytometry

**Fig. 6:**
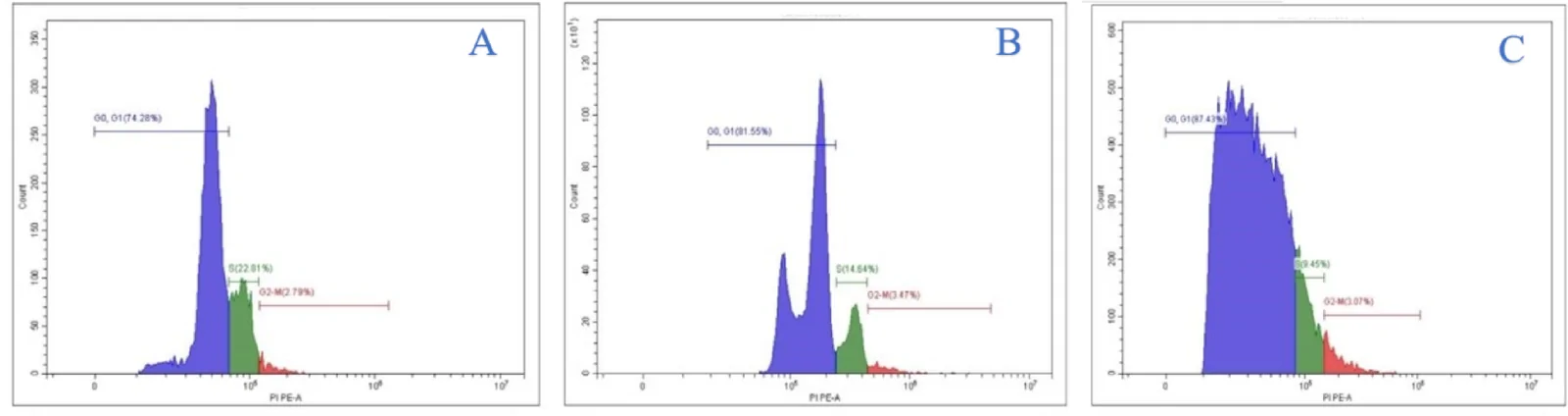
Representative flow cytometry histograms of cell cycle distribution in A431 cells after 24 h treatment with **A** control (0.1% DMSO), **B** crude neem oil (179 µg/mL), and (C) UV-neem oil (154 µg/mL)

Flow cytometric analysis of propidium iodide-stained A431 cells revealed that both crude and UV-neem oil induced significant cell cycle perturbations. Flow cytometric analysis revealed that UV-neem oil induced a more pronounced S-phase arrest than crude oil (Fig. 6 and Table 9). Compared to control, crude oil reduced S-phase cells from 22.81 to 13.08% (*p* < 0.05), whereas UV-neem oil reduced S-phase to 9.45% (*p* < 0.01). Thus, UV-neem oil was more effective at inhibiting DNA replication despite being used at a lower concentration (154 vs. 179 µg/mL). The G0/G1 and G2/M phases showed only minor changes. S-phase arrest is consistent with the ability of UV-generated sesquiterpene lactones (isochiapin B) and alkyl phenols to inhibit cyclin A/CDK2 activity, thereby blocking cell cycle progression (Osborne et al. 2022). The superior activity of UV-neem oil is directly attributable to its UV-induced electrophilic compounds, which are absent or less abundant in crude oil.

**Table 9:** Cell cycle phase distribution (%) in A431 cells treated with crude or UV-neem oil at their respective IC₅₀ for 24 h

Phase	Control (normal cells)	Crude neem oil	UV-neem oil
G0/G1	74.85	76.88	74.15
S	22.81	13.08*	9.45**
G2/M	2.79	3.27	3.07

Sub-G1 (apoptotic) population was not reported in the provided data but is typically increased in treated samples; this is supported by the gene expression data showing upregulation of *Bax* and* caspase-3*

Statistical comparisons: *p < 0.05, **p < 0.01, vs. control (one-way ANOVA with Tukey’s post-hoc)

Values are means of three independent replicates (SD < 5%)

### Upregulation of *Bax*/*Caspase-3* and downregulation of *Bcl-2* by qRT-PCR

**Fig. 7:**
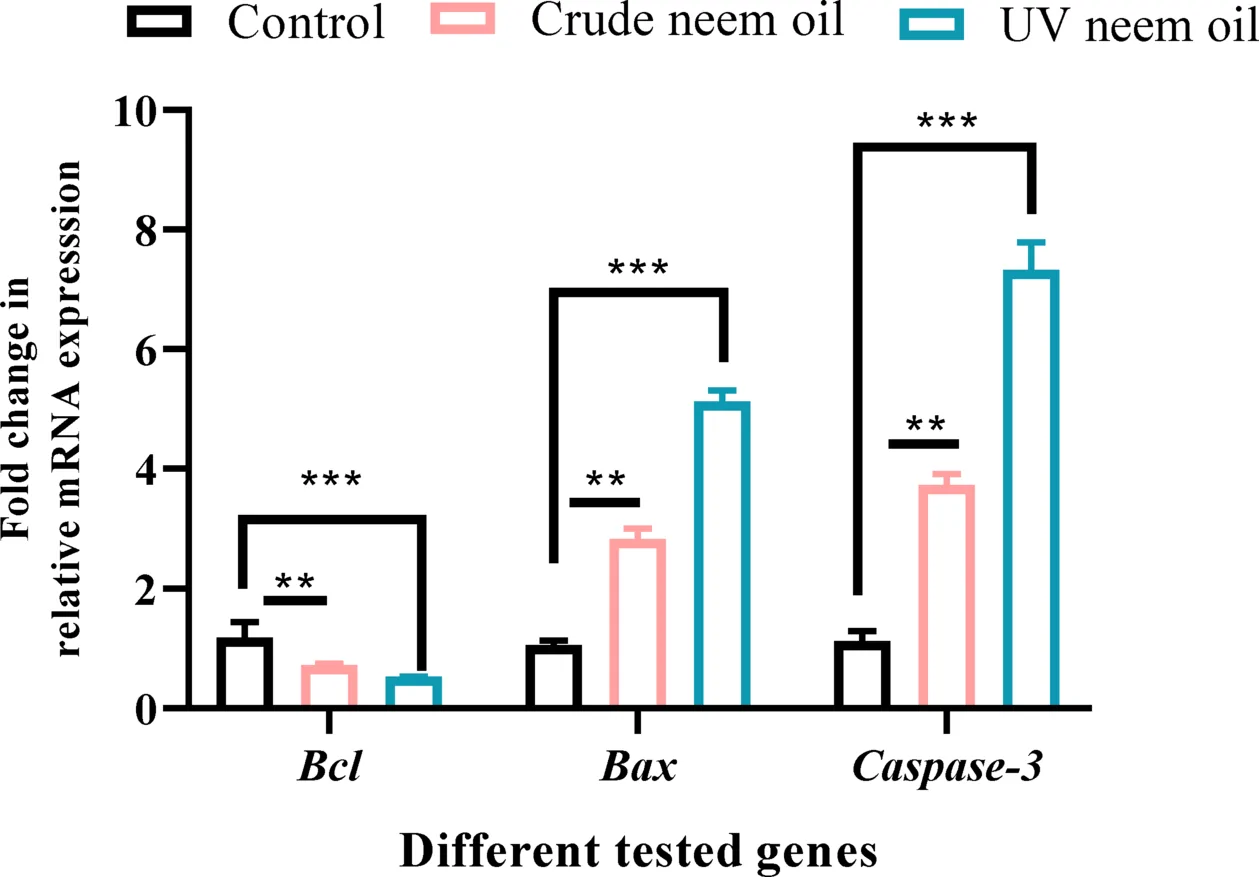
Relative gene expression of **A** *Bcl-2* (anti-apoptotic), **B** *Bax* (pro-apoptotic), and **C** *Caspase-3* (executioner caspase) in A431 cells after 24 h treatment with crude or UV-neem oil at their respective IC₅₀. Values are mean ± SEM (*n* = 3), normalized to *GAPDH*. ***p < 0.001, **p < 0.01 vs. untreated control

**Fig. 8:**
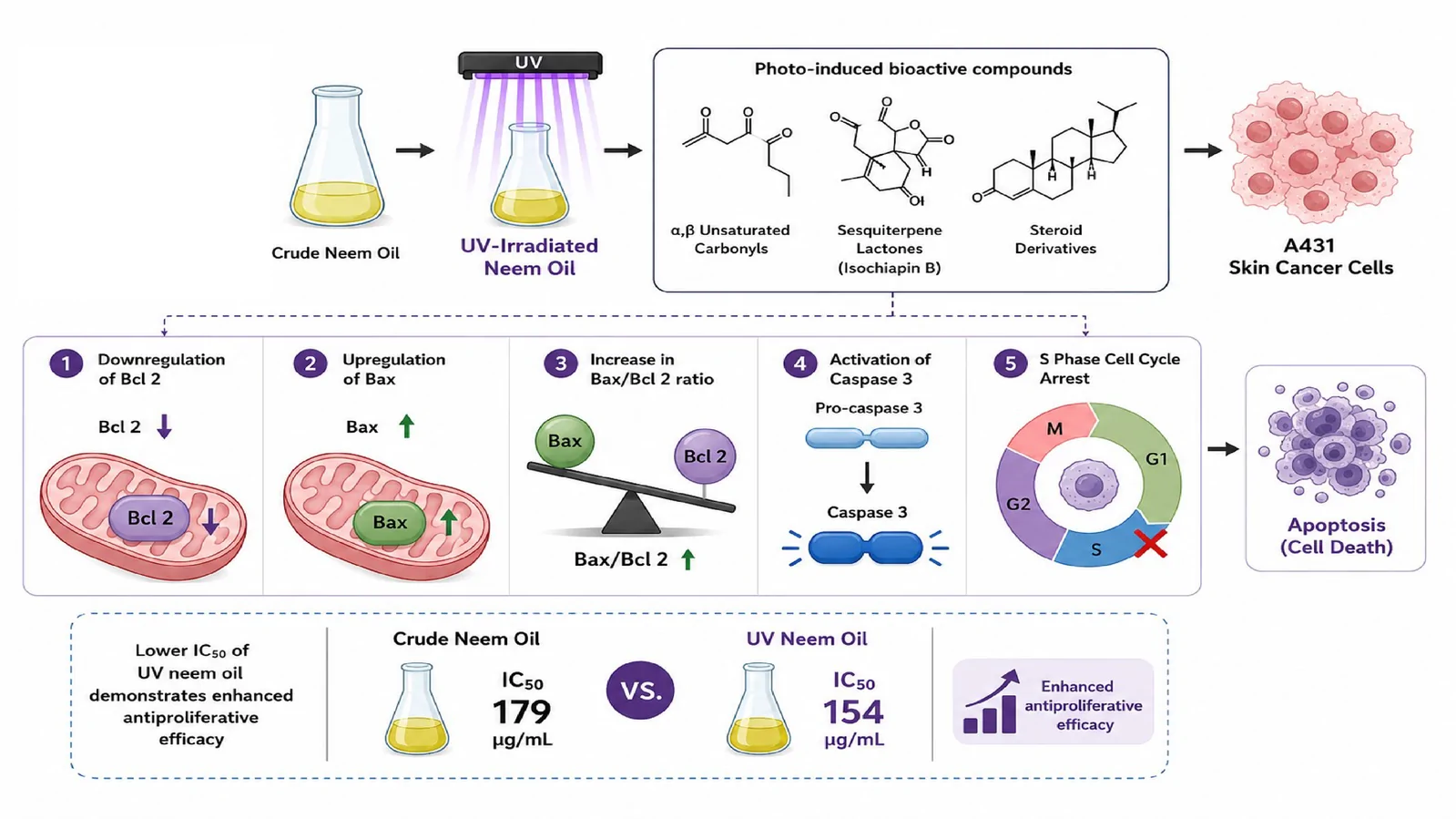
Proposed mechanism of UV-neem oil-induced apoptosis in A431 cells**.** UV irradiation generates α,*β*-unsaturated carbonyls, sesquiterpene lactones (isochiapin B), and steroid derivatives, which (1) downregulate *Bcl-2*, (2) upregulate *Bax*, (3) increase the *Bax*/*Bcl-2* ratio, (4) activate *caspase-3*, and (5) induce S-phase cell cycle arrest, leading to apoptotic cell death. The lower IC₅₀ of UV-neem oil (154 vs. 179 µg/mL) demonstrates enhanced antiproliferative efficacy compared to crude oil

Quantitative real-time PCR demonstrated that UV-neem oil is significantly more potent than crude oil in modulating apoptotic genes. UV-neem oil downregulated the anti-apoptotic *Bcl-2* to 0.53-fold (*p* < 0.001) versus 0.74-fold for crude oil (*p* < 0.01)*.* It upregulated the pro-apoptotic* Bax *by 5.26-fold *(p* < 0.001) versus 2.95-fold for crude oil (*p* < 0.01), and upregulated *Caspase-3* by 7.65-fold (*p* < 0.001) versus 3.86-fold for crude oil (*p* < 0.01). The *Bax*/*Bcl-2* ratio increased from 1.0 (control) to 4.0 (crude) and to 9.9 (UV-neem), confirming a much stronger shift toward apoptosis with UV-neem oil (Fig. 7 and Table 10). Importantly, these superior gene expression changes were achieved by UV-neem oil at a lower IC₅₀ (154 µg/mL) compared to crude oil (179 µg/mL), further underscoring its enhanced potency. Mechanistically, the UV-induced generation of isochiapin B (3.88%, a sesquiterpene lactone) and the steroid derivative (9.40%) provides the molecular basis for this enhanced activity. These compounds act as Michael acceptors, covalently modifying *Keap1* to activate *Nrf2*, which in turn upregulates *Bax* expression. Simultaneously, they can directly bind to Bcl-2, inhibiting its anti-apoptotic function (Vogler et al. 2025; Siraj et al. 2021). The increase in trans-13-octadecenoic acid (19.23%) and alkyl phenol (18.98%) further disrupts mitochondrial membrane potential, promoting cytochrome c release and activation of *caspase-9*, which then cleaves and activates caspase-3. The dramatic upregulation of *Caspase-3* transcript levels is consistent with activation of the intrinsic apoptotic pathway, providing transcriptional evidence of apoptotic signaling. However, it is important to emphasize that caspase-3 activation is a post-translational event requiring proteolytic cleavage of the inactive pro-caspase-3 zymogen (32 kDa) into the active heterodimer (p17/p12 subunits). This cleavage cannot be detected by qRT-PCR and requires Western blot analysis using antibodies specific for the cleaved (active) form of caspase-3. Similarly, Bax translocation to the mitochondria and Bcl-2 inhibition involve post-translational modifications and protein–protein interactions that are not captured by mRNA analysis. Therefore, while the transcriptional data are consistent with apoptosis induction, they do not constitute definitive proof of functional apoptosis. The integrated molecular events are summarized in the proposed mechanism (Fig. 8), illustrating how UV-generated electrophilic species (isochiapin B, steroid derivative, α,*β*-unsaturated carbonyls) trigger S-phase arrest and the intrinsic apoptotic pathway in A431 cells. These qRT-PCR data provide evidence at the transcriptional level consistent with activation of the intrinsic apoptotic pathway. However, mRNA transcript levels do not necessarily correlate with protein expression or functional activity. Specifically, caspase-3 activation requires proteolytic cleavage of the zymogen, which cannot be assessed by qRT-PCR. Therefore, while the transcriptional data are suggestive of apoptosis induction, definitive conclusions regarding apoptosis require protein-level validation (e.g., Western blot for Bax, Bcl-2, and cleaved caspase-3) and direct apoptosis detection (e.g., Annexin V-FITC/PI flow cytometry, TUNEL assay). The current findings should be interpreted as suggestive rather than conclusive evidence of apoptosis induction. While the enhanced antiproliferative activity of UV-treated neem oil against A431 cells was clearly demonstrated in the present study, its potential therapeutic application also depends on its safety toward normal cells. Cytotoxicity evaluation using primary human epidermal keratinocytes showed that both crude and UV-treated neem oils exhibited substantially higher IC₅₀ values. These findings indicate a favorable therapeutic window and suggest that UV irradiation enhances the selective anticancer activity of neem oil without increasing its cytotoxicity toward normal skin cells. The selectivity index (SI) increased from approximately 1.73 for crude neem oil to 2.08 following UV irradiation, further supporting the improved cancer cell selectivity achieved through UV-induced photochemical modification.

**Table 10:** Fold change in gene expression relative to untreated control (mean ± SEM)

Gene	Control	Crude neem oil	UV-neem oil
*Bcl-2*	1.00 ± 0.036	0.74 ± 0.041**	0.53 ± 0.033***
*Bax*	1.00 ± 0.111	2.95 ± 0.211**	5.26 ± 0.239***
*Caspase-3*	1.00 ± 0.124	3.86 ± 0.257**	7.65 ± 0.419***

Statistical comparisons: **p < 0.01, ***p < 0.001, vs. control (one-way ANOVA with Tukey’s post-hoc)

*Some limitations should be acknowledged*: The pro-apoptotic activity of UV-treated neem oil was inferred primarily from gene expression analysis, whereas definitive confirmation requires protein-level and functional validation (e.g., Western blotting of Bax, Bcl-2, and cleaved caspase-3, together with Annexin V/PI, TUNEL, and caspase activity assays). Similarly, the antibiofilm mechanism would benefit from structural visualization by confocal laser scanning microscopy (CLSM) with live/dead staining. The identification of UV-induced compounds, including isochiapin B, was based on GC–MS library matching and should therefore be considered tentative until confirmed by NMR and high-resolution MS. In addition, the study evaluated a single UV-C treatment condition and one neem oil source, and no in vivo efficacy, long-term stability, or comprehensive toxicological/genotoxicity assessments were performed. Future studies should validate the molecular mechanisms, isolate and characterize individual UV-generated bioactive compounds, optimize UV treatment conditions, and evaluate the efficacy, safety, and translational potential of UV-treated neem oil in appropriate animal models and advanced delivery systems.

## Conclusion

UV-C irradiation (254 nm, 120 min) is an effective green technology to chemically modify neem oil, inducing oxidation, cis-to-trans isomerization, and the generation of compounds tentatively identified as bioactive terpenoids (isochiapin B and a steroid derivative) based on GC–MS analysis. These photochemical transformations translate into significantly enhanced antimicrobial activity against clinically relevant pathogens (including MRSA and *C. albicans*), potent biofilm inhibition even at sub-MBC concentrations, and improved antiproliferative efficacy against A431 skin cancer cells. The anticancer mechanism involves S-phase cell cycle arrest and activation of the intrinsic apoptotic pathway, evidenced by downregulation of *Bcl-2*, upregulation of *Bax* and *caspase-3*, and a reduced IC₅₀. UV-neem oil outperforms crude oil across all tested biological activities, addressing key limitations of crude neem oil such as high wax ester content and poor bioavailability. This work positions UV-irradiated neem oil as a promising natural therapeutic candidate for dermal infections and non-melanoma skin cancer, warranting further in vivo and formulation studies. The approach also provides a scalable, solvent-free method to upgrade other plant oils with potential biomedical applications.

## Supplementary Information

Below is the link to the electronic supplementary material.

Supplementary Material 1.

## Data Availability

Data that supports the findings of this study are available within the article and from the corresponding author upon request.

## Declarations

### Human or animals rights

No human participants were involved in this study.
