# Supplementary material for: Mechanistic investigation of UV-irradiated neem oil against microbial biofilms and A431 skin cancer cells via Bax/Bcl2/caspase-3 modulation
Source: Bioresour Bioprocess. 2026 Sep 15;13(1):136. doi: 10.1186/s40643-026-01111-7 (PMC13578163; doi:10.1186/s40643-026-01111-7)
Supplement: Supplementary file 1 [file 40643_2026_1111_MOESM1_ESM.docx]

**Supplementary Table S1. MBC/MFC values and corresponding sublethal concentrations (25%, 50%, and 75% of MBC/MFC) of crude and UV-treated neem oils used in the antibiofilm assays against the tested microorganisms.**

| Microbes | Crude oil MBC/MFC (µg/mL) | | | | UV treated oil MBC/MFC (µg/mL) | | | |
| --- | --- | --- | --- | --- | --- | --- | --- | --- |
|  | MBC/MFC | 25 % | 50 % | 75 % | MBC/MFC | 25 % | 50 % | 75 % |
| *B. subtilis* | 62.5 | 15.63 | 31.25 | 46.88 | 31.25 | 7.81 | 15.63 | 23.44 |
| MRSA | 250 | 62.50 | 125.00 | 187.50 | 31.25 | 7.81 | 15.63 | 23.44 |
| *E. coli* | 125 | 31.25 | 62.50 | 93.75 | 15.62 | 3.91 | 7.81 | 11.72 |
| *P. vulgaris* | 62.5 | 15.63 | 31.25 | 46.88 | 31.25 | 7.81 | 15.63 | 23.44 |
| *C. albicans* | 125 | 31.25 | 62.50 | 93.75 | 31.25 | 7.81 | 15.63 | 23.44 |
